# Integration of the Human Gut Microbiome and Serum Metabolome Reveals Novel Biological Factors Involved in the Regulation of Bone Mineral Density

**DOI:** 10.3389/fcimb.2022.853499

**Published:** 2022-03-16

**Authors:** Jonathan Greenbaum, Xu Lin, Kuan-Jui Su, Rui Gong, Hui Shen, Jie Shen, Hong-Mei Xiao, Hong-Wen Deng

**Affiliations:** ^1^ Tulane Center of Biomedical Informatics and Genomics, Deming Department of Medicine, Tulane University School of Medicine, Tulane University, New Orleans, LA, United States; ^2^ Department of Endocrinology and Metabolism, The Third Affiliated Hospital of Southern Medical University, Guangzhou, China; ^3^ Center of Systems Biology, Data Information and Reproductive Health, School of Basic Medical Science, Central South University, Changsha, China

**Keywords:** metagenomics, metabolomics, data integration, osteoporosis, bone

## Abstract

While the gut microbiome has been reported to play a role in bone metabolism, the individual species and underlying functional mechanisms have not yet been characterized. We conducted a systematic multi-omics analysis using paired metagenomic and untargeted serum metabolomic profiles from a large sample of 499 peri- and early post-menopausal women to identify the potential crosstalk between these biological factors which may be involved in the regulation of bone mineral density (BMD). Single omics association analyses identified 22 bacteria species and 17 serum metabolites for putative association with BMD. Among the identified bacteria, *Bacteroidetes* and *Fusobacteria* were negatively associated, while *Firmicutes* were positively associated. Several of the identified serum metabolites including 3-phenylpropanoic acid, mainly derived from dietary polyphenols, and glycolithocholic acid, a secondary bile acid, are metabolic byproducts of the microbiota. We further conducted a supervised integrative feature selection with respect to BMD and constructed the inter-omics partial correlation network. Although still requiring replication and validation in future studies, the findings from this exploratory analysis provide novel insights into the interrelationships between the gut microbiome and serum metabolome that may potentially play a role in skeletal remodeling processes.

## Introduction

Osteoporosis is a progressive age-related condition associated with reduced bone mineral density (BMD) and increased susceptibility to low trauma fractures, which are the clinical endpoint of the disease. It represents the most prevalent metabolic bone disorder affecting >200 million people worldwide (Reginster and Burlet, 2006), and the burden is particularly large among postmenopausal women, which is mainly attributed to the reduced production of estrogen and other hormonal/metabolic changes that occur during menopause. It is estimated that at least one in three postmenopausal women have osteoporosis, and nearly half of those women will experience fragility fractures in their remaining lifetime (Melton et al., 1992). Dual-energy X-ray absorptiometry (DXA) derived BMD measurements of the hip and spine are the most frequently used metric for clinically diagnosing osteoporosis, as well as the most powerful known risk factor for predicting fracture risk (Kanis et al., 2005).

The gut microbiome, composed of the bacteria residing in the human gastrointestinal tract, is involved in a variety of diverse functions that are important for physiological wellbeing. There are several potential mechanisms through which the microbiome may impact bone metabolism, as previously reviewed (Hernandez et al., 2016; Chen et al., 2017). The microbiota can influence the intestinal absorption of essential minerals (e.g., calcium) that are important for maintaining skeletal homeostasis (Weaver, 2015), elicit immune responses which may alter the levels of inflammatory cytokines (e.g., TNF-*α*) that are important for bone health (Sjogren et al., 2012), produce metabolic byproducts (e.g., short chain fatty acids) which regulate critical cell signaling factors for bone remodeling processes (Lucas et al., 2018), and modulate the levels of hormones and neurotransmitters through the gut-brain axis (Cryan et al., 2019), including some (e.g., serotonin) that have been shown to interact with bone cells (Bliziotes, 2010). Although experimental animal models have provided compelling evidence that the gut microbiome may play a role in the regulation of bone mass (Sjogren et al., 2012), only a few limited studies have explored this relationship in humans (Wang et al., 2017; Das et al., 2019; Xu et al., 2020). While these early efforts reported significant differences in the microbial diversity between osteoporosis cases and healthy controls, they were generally limited by small sample sizes and the inability to reveal specific trait-associated bacteria or functional mechanisms.

Metabolomics enables the comprehensive profiling of the intermediate and end products of cellular metabolism. Since metabolites represent the downstream expression of genomic, transcriptomic, and proteomic factors, small changes in other omics may be amplified at the metabolomic level, enabling the detection of critical biomarkers or corresponding therapeutic target pathways closely related to disease risk (Johnson et al., 2016). However, the application of metabolomics for osteoporosis is rather limited. At present, most efforts are confined to animal experiments (Lv et al., 2016), although several early studies in humans have identified novel osteoporosis biomarkers involved in the metabolism of tryptophan, phenylalanine, lipids, and energy (Miyamoto et al., 2018; Moayyeri et al., 2018; Zhao Q et al., 2018; Gong et al., 2021). Notably, some studies demonstrated that the effects of the novel metabolites identified were more significant than classical bone turnover markers (Qi et al., 2016), supporting the crucial functions of small molecule metabolites in BMD regulation and osteoporosis prediction.

It is well established that changes in diet may be accompanied by shifts in the composition of the microbiome (Singh et al., 2017), but perhaps even more important is the resulting effect on the human metabolome. The diet contains many compounds that cannot be broken down by human digestive enzymes, and therefore pass to the gut where they are catabolized by the microbiota (Lamichhane et al., 2018). Some of the metabolic byproducts generated during these processes may then be absorbed into the circulating blood, where they can potentially impact human health. For instance, the gut metabolite Trimethylamine N-oxide (TMAO), regulated by dietary phosphatidylcholine intake, has been shown to promote the development of atherosclerosis (Wang et al., 2011; Tang et al., 2013). Based on these findings, a novel therapeutic approach was established to inhibit microbial production of TMAO (Wang et al., 2015). We hypothesized that there could be similar undiscovered mechanisms which contribute to the osteoporosis susceptibility.

**Figure 1:**
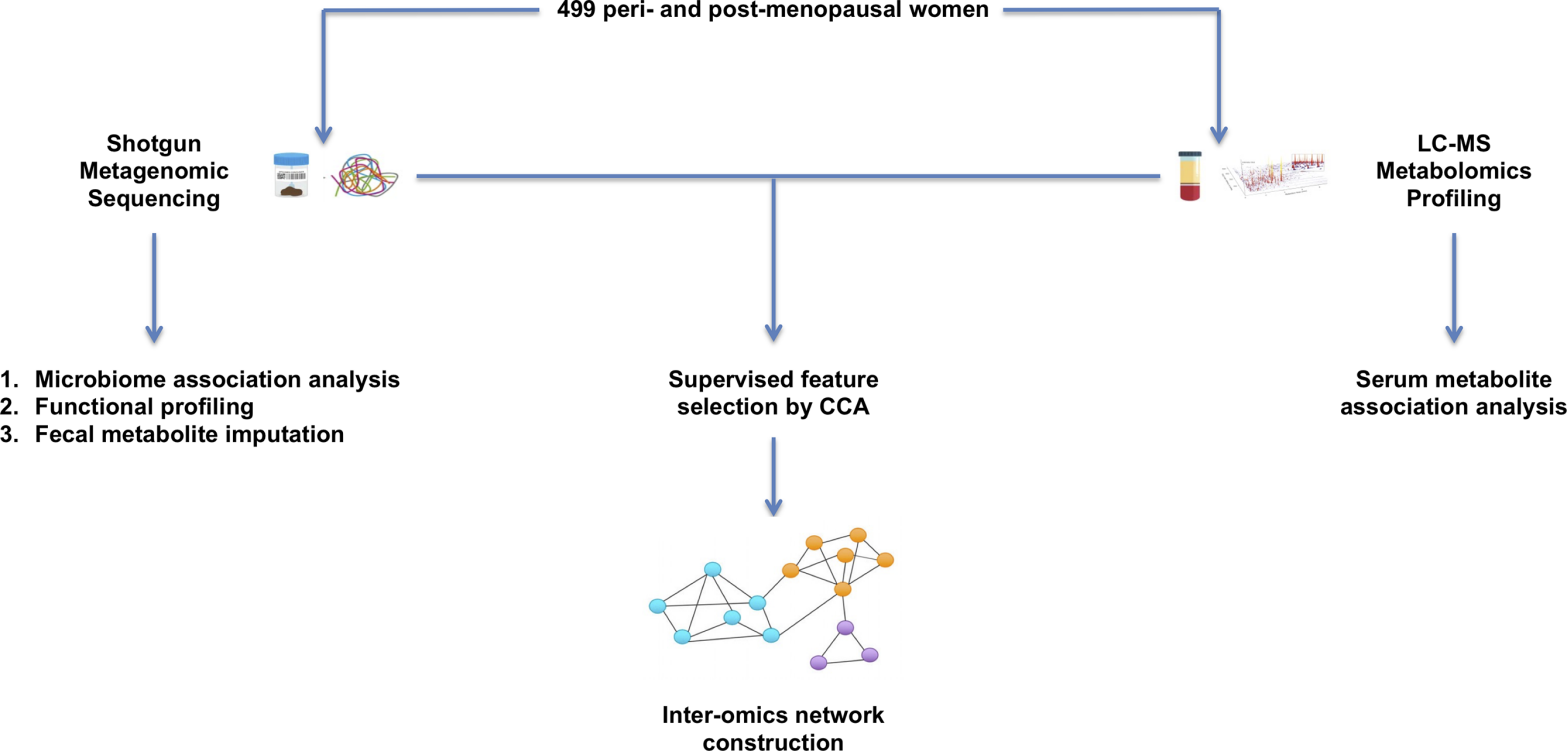
Overview of study workflow. 499 peri- and early post-menopausal women provided stool and blood samples for shotgun metagenomic sequencing and untargeted serum metabolomics profiling. Single omics association analyses were first conducted to identify microbes and metabolites that are associated with BMD. The paired microbiome and metabolite profiles were then integrated by performing a supervised feature selection with respect to BMD. The selected features were used to conduct inter-omics network analysis to explore the crosstalk between these biological factors.

Multi-omics integration analyses of microbiome and metabolite profiles collected from the same individuals are very much needed to elucidate the full range of interactions between these biological factors with respect to bone phenotypes. We integrated the paired gut microbiome and untargeted serum metabolite profiles from a large sample of peri- and early post-menopausal Chinese women to explore the crosstalk which may contribute to BMD variation at the femoral neck, the most common site for hip fracture, which is one of the most devastating types of osteoporotic fractures (LeBlanc et al., 2014). An overview of the study workflow is provided in **Figure 1**.

## Materials and Methods

### Sample Recruitment

We randomly recruited 499 peri- and early post-menopausal Chinese women (aged 40 – 65) living in Guangzhou City, China. Perimenopausal refers to the menopause transition phase, characterized by irregular menstrual cycles, while postmenopausal is defined by the cessation of menstrual periods for >1 year (Lumsden, 2016). Women who had taken antibiotics or estrogens within three months of enrollment were excluded. We also excluded women with preexisting conditions relevant to bone mass development such as serious residual effects from cerebral vascular disease, diabetes mellitus, chronic renal failure, chronic liver failure, chronic lung disease, alcohol abuse, corticosteroid therapy for more than 6 months duration, evidence of other metabolic or inherited bone disease, rheumatoid arthritis, collagen disorders, and chronic gastrointestinal diseases. Each subject signed an informed consent, and the study protocol was approved by the Medical Ethics Committee of Southern Medical University.

BMD of the hip and spine were measured with DXA (Lunar, GE Healthcare, Madison, WI, USA) by trained and certified research staff. The machine was calibrated daily using a phantom scan for quality assurance, and the accuracy of BMD measurement was assessed by the coefficient of variation for repeated measurements, which was 0.89% for spine BMD. To minimize information loss from artificially dichotomizing individuals into low/high BMD groups, BMD was considered as a quantitative trait. BMD measurements were standardized to have a mean of zero and standard deviation of one, and the normalized values were used as the phenotype.

Each subject provided stool and blood samples for metagenomic and metabolomics analyses, respectively. Stool samples were frozen at -80°C after sample procurement until DNA extraction. To avoid variation due to circadian rhythm, which is known to affect the metabolome (Sahar and Sassone-Corsi, 2012), 10 ml of blood was drawn from each subject after >8 hours of overnight fasting. Serum was extracted from the blood samples according to the protein precipitation protocol (Bruce et al., 2009) developed for metabolomics analysis, aliquoted, and stored at -80°C until used for further analysis. The subjects also completed a questionnaire to collect relevant covariate information (e.g., demographic and lifestyle factors). Since sex hormones are involved in metabolism in general (Guarner-Lans et al., 2011), and bone metabolism more specifically (Drake et al., 2013), the serum levels of follicle stimulating hormone (FSH) and estradiol were measured using routine enzyme linked immunoassay ELISA kits (Immunodiagnostic Systems, Gaithersburg, MD, USA).

### Metagenomic Sequencing

DNA was extracted from 200 mg of stool sample using the E.Z.N.A.^®^ Stool DNA Kit (Omega, Norcross, GA, USA) following the manufacturer’s protocol. The total DNA was eluted in 50 *μ*l of elution buffer (QIAGEN, Hilden, Germany) and stored at -80°C until metagenomic sequencing (LC-BIO Technologies Co. LTD., Hang Zhou, China). We constructed a fecal DNA library, and used HiSeq 4000 (Illumina, San Diego, CA, USA) with the paired end 150 bp strategy to conduct sequencing. Fecal DNA was fragmented using dsDNA Fragmentase (New England BioLabs, Ipswich, MA, USA) by incubating at 37°C for 30 min, and the DNA library was constructed by TruSeq Nano DNA LT Library Preparation Kit (Illumina, San Diego, CA, USA). Blunt-end DNA fragments were generated using a combination of fill-in reactions and exonuclease activity, and size selection was performed with the provided sample purification beads. An A-base was added to the blunt ends of the strands, preparing them for ligation to the indexed adapters. Each adapter contained a T-base overhang for ligating the adapter to the A-tailed fragmented DNA. The adapters were ligated to the fragments and the ligated products were amplified with PCR by the following conditions: initial denaturation at 95°C for 3 min, 8 cycles of denaturation at 98°C for 15 sec, annealing at 60°C for 15 sec, extension at 72°C for 30 sec, and then final extension at 72°C for 5 min.

The raw sequencing reads were then processed to obtain valid reads for further analysis by removing sequencing adapters with cutadapt v1.9 (Martin, 2011), trimming low quality reads using fqtrim v0.94 (Pertea, 2015), and aligning reads to the human reference genome (GRCh38/hg38) to remove host contamination with Bowtie2 v2.2.0 (Langmead and Salzberg, 2012). The quality filtered reads were *de novo* assembled to construct the metagenome for each sample using SPAdes v3.10.0 (Bankevich et al., 2012). All coding regions of metagenomic contigs were predicted using MetaGeneMark v3.26 (Zhu et al., 2010), and the coding sequences of all samples were clustered to obtain UniGenes with CD-HIT v4.6.1 (Fu et al., 2012). The UniGene abundances for a given sample were estimated by transcripts per million (TPM) based on the number of aligned reads, ${\text{G}}_{\text{k}}=\frac{{\text{r}}_{\text{k}}}{{\text{L}}_{\text{k}}}*\frac{1}{\Sigma_{\text{i}=1}^{\text{n}}\frac{\Gamma_{\text{i}}}{{\text{L}}_{\text{i}}}}*10^{6}$ where *k* refers to the *k^th^* UniGene, *r* is the number of UniGene reads, and *L* is the UniGene length.

The DIAMOND+MEGAN approach was then applied for taxonomic annotation. The UniGenes were aligned against the NCBI non-redundant protein database with DIAMOND v0.9.20 (Buchfink et al., 2015). The quality of the alignments was determined based on the bit score, which represents the required size of a sequence database in which the current match could be found by chance, and *E*-value, which denotes the likelihood that a given sequence match is due purely to chance. The resulting alignments were then used as input for taxonomic binning using the lowest common ancestor (LCA) algorithm in MEGAN v6.12.3 (Huson et al., 2007), which places a read on the lowest taxonomic node in the NCBI taxonomy that lies above all taxa to which the read has a significant alignment. We note that while the limitations of this local sequence alignment approach have been documented (Koski and Golding, 2001), it is a standard protocol for taxonomic profiling (Bagci et al., 2021).

The microbiome data are relative abundances since the total number of read counts per sample is highly variable and constrained by the maximum number of reads the sequencer can provide (Gloor et al., 2017), and the data are considered compositional because the relative abundances of all bacteria species within each sample are proportions which have a unit sum. We eliminated the rare species with an average relative abundance <0.01% to reduce the extreme sparsity of the data and remove sequencing artifacts (Cao et al., 2020). The relative abundances were then normalized by the centered log-ratio (CLR) transformation, which has been shown to be effective in transforming the compositional data to be approximately multivariate normal (Gloor et al., 2017).

### Serum Metabolomics Profiling

The serum samples were thawed on ice, and metabolites were extracted with 50% methanol buffer. 20 μl of sample was extracted with 120 μl of precooled 50% methanol, vortexed for 1 min, incubated at room temperature for 10 min, and stored overnight at –20°C. After centrifugation at 4,000g for 20 min, the supernatants were transferred into new 96-well plates and stored at –80°C prior to the liquid chromatography mass spectrometry (LC-MS) metabolomics analysis (LC-BIO Technologies Co. LTD., Hang Zhou, China). Pooled quality control samples were prepared by combining 10 μl of each extraction mixture. All chromatographic separations were performed using an ultra-performance liquid chromatography (UPLC) system (SCIEX, UK), and an ACQUITY UPLC BEH Amide column (100mm*2.1mm, 1.7μm, Waters, Wilmslow, UK) was used for the reversed phase separation. The column oven was maintained at 35°C, the flow rate was set to 0.4 ml/min, and the mobile phase consisted of solvent A (25mM ammonium acetate+25 mM NH4H2O) and solvent B (IPA: ACN=9:1+0.1% formic acid). Gradient elution conditions were set as follows: 0 ~ 0.5 min, 95% B; 0.5 ~ 9.5 min, 95% ~ 65% B; 9.5 ~ 10.5 min, 65% ~ 40% B; 10.5 ~ 12 min, 40% B; 12 ~ 12.2 min, 40% ~ 95% B; 12.2 ~ 15 min, 95% B.

A high-resolution tandem mass spectrometer Triple TOF5600plus (SCIEX, UK) was used to detect the metabolites eluted from the column in both positive and negative ion modes. The curtain gas was set to 30 PSI, ion source gas one and two were both set to 60 PSI, and the interface heater temperature was set to 650°C. The Ionspray voltage floating was 5000 V for positive ion mode and –4500 V for negative ion mode. The mass spectrometry data were acquired in IDA mode, and the TOF mass range was from 60 to 1200 Da. The survey scans were acquired in 150 ms, and as many as 12 product ion scans were collected if exceeding a threshold of 100 counts per second and with a 1+ charge-state. The total cycle time was fixed to 0.56 s. Four different time bins were summarized for each scan at a pulser frequency value of 11 kHz through monitoring of the 40 GHz multi-channel TDC detector with four-anode/channel detection. Dynamic exclusion was set for 4 s. During the acquisition, the mass accuracy was calibrated every 20 samples. To evaluate the stability of the LC-MS during the whole acquisition, a pooled quality control sample was acquired after every 10 samples.

The acquired MS data pretreatments including peak picking, peak grouping, retention time correction, and second peak grouping were performed using XCMS v3.16.1 (Smith et al., 2006). CAMERA v1.50 (Kuhl et al., 2012) was used to annotate the identified features with related isotopic peaks and adducts. Each ion was characterized by retention time and mass-to-charge ratios (m/z), and the intensities of each peak were recorded. Metabolite identification and data processing were performed using metaX v1.0.3 (Wen et al., 2017). The Human Metabolome Database (HMDB) and Kyoto Encyclopedia of Genes and Genomes (KEGG) were used to annotate metabolites by performing a mass-based search with a weight tolerance of 10 ppm. To provide more confident and reproducible study findings, we retained metabolites with annotations that were validated using an in-house fragment spectrum library.

Metabolite features detected in <50% of quality control samples or <80% of biological samples were removed, and the remaining peaks with missing values were imputed with the k-nearest neighbor algorithm. Probabilistic quotient normalization was applied to minimize technical artifacts, and robust spline correction was used for the post-acquisition correction of batch effects. In addition, the relative standard deviations of the metabolite features were calculated across all quality control samples, and those >30% were removed. The remaining metabolite features were log transformed and scaled to have zero mean and unit variance, which is a common normalization technique (Zhao Q et al., 2018; Gong et al., 2021). The log transformation converts skewed data to symmetric, while scaling makes all metabolites of equal importance and enables comparison based on correlations (Li et al., 2016).

### Microbiome Association Analysis

Individual microbes were tested for association with BMD using a constrained elastic net regression model, which is a commonly used feature selection approach with compositional covariates (Lin et al., 2014). The model imposes a sparsity penalty along with a constraint that the regression coefficients of the CLR-transformed relative abundances sum to zero, $\hat{\beta }=\arg\min(\|\text{y}-\text{X}\beta {\|}^{2}+\lambda_{1}\|\beta {\|}_{1}+\lambda_{2}\|\beta {\|}^{2})$ subject to $\Sigma_{\text{i}=1}^{\text{s}}\beta_{\text{i}}=0$ . The elastic net regularization is a combination of both ridge and lasso penalty functions, where ridge results in a nonzero coefficient for every feature and lasso only assigns nonzero coefficients to the most strongly associated features. Since the penalized regression model does not provide conventional association *p*-values, partial Spearman correlation analysis was used to individually test each microbe selected in the initial feature screening.

### Functional Profiling of Microbiota

The abundances of metabolic pathways in the microbiome community were profiled using the Human Microbiome Project Unified Metabolic Analysis Network (HUMAnN2) pipeline (Franzosa et al., 2018). HUMAnN2 first maps metagenomic reads to the pangenomes (Huang et al., 2014) of species identified by taxonomic profiling. The protein-coding sequences in these pangenomes have been pre-annotated to their respective UniRef90 families (Suzek et al., 2015), which serve as a non-redundant protein sequence database. Metagenomic reads that do not align to a known pangenome are subjected to a translated search against the full UniRef90 database. All hits are weighted by quality and sequence length to estimate the gene abundances. These genes are then annotated to metabolic enzymes and further analyzed to quantify the abundances of complete metabolic pathways obtained from MetaCyc (Caspi et al., 2016). HUMAnN2 assigns a coverage and abundance score for each pathway in each sample based on the detection of all its constituent genes. The coverage and abundance scores represent the number and abundance of complete copies of the pathway in each sample. Partial Spearman correlation analyses were used to test the associations between the pathway abundances and BMD.

### Fecal Metabolite Imputation

The Model-based Genomically Informed High-dimensional Predictor of Microbial Community Metabolic Profiles (MelonnPan) approach (Mallick et al., 2019) was applied to predict the abundances of fecal metabolites from the microbiome gene abundances estimated by HUMAnN2. Elastic net prediction models were trained to select a sparse set of microbiome genes that are predictive for each fecal metabolite based on an independent set of 155 reference subjects for which both metagenomic and metabolomic profiling of the stool samples were both available (Franzosa et al., 2019). The fecal metabolite concentrations were then imputed as a linear combination of the microbiota gene abundances with weights learned from the training set. We retained the well predicted fecal metabolites, which had at least a moderate correlation (Spearman ρ >0.3) between the observed and imputed metabolite abundances in the training sample, as previously detailed (Mallick et al., 2019).

### Metabolite Association Analysis

Partial least squares regression (PLS) is a multivariate approach which combines aspects of principal component analysis (PCA) and linear regression (Rohart et al., 2017). The principle is to extract a set of orthogonal components that have large covariance with the phenotype. PLS is well suited for the metabolomics analysis due to the high degree of correlation between functionally related metabolites (i.e., metabolites involved in the same metabolic pathways). A variable importance in projection (VIP) score is used to summarize the contribution of each feature to the model, which is computed as a weighted sum of the squared correlations between the PLS components and phenotype. Metabolites with VIP ≥2.0 were considered important for the phenotype. As a complementary approach, all metabolites were also individually tested using linear regression.

### Coinertia Analysis

The global similarity between the gut microbiome and serum metabolome was investigated using coinertia analysis, which identifies successive axes of covariance between two datasets measured on a single group of subjects (Dray and Dufour, 2007). Principal coordinate analysis (PCoA) with Bray Curtis distance and PCA were applied to the microbiome and metabolite profiles, respectively, and the ordinations were used as input for the coinertia analysis. The coinertia analysis produces an RV coefficient, which is a multivariate extension of the squared Pearson correlation coefficient computed as $\text{RV}=\frac{\text{coinertia (X,Y)}}{\sqrt{\text{coinertia (X,X)}}\sqrt{\text{coinertia (Y,Y)}}}$ where 0 < RV < 1. The coinertia between two hyperspaces is defined as the sum of the squared covariances between all variable pairs. Statistical significance of the RV coefficient was determined through a Monte-Carlo permutation test.

### Supervised Multi-Omics Feature Selection

Canonical correlation analysis (CCA) has previously been proposed as a promising approach for performing integration analysis (Parkhomenko et al., 2009). Assuming two different data modalities measured on the same subjects, CCA seeks weighted linear combinations of the features from each dataset that have large correlation. However, the conventional CCA model assigns nonzero weights to every feature, which can result in overfitting for high dimensional data, and CCA is traditionally unsupervised since it does not take the phenotype information into consideration.

The overfitting issue can be addressed by introducing a sparsity penalty into the CCA model, which allows for the incorporation of feature selection. The sparse CCA model can then be further extended to be supervised (sCCA), such that the selected features are correlated across omics modalities with importance for a quantitative phenotype (Parkhomenko et al., 2009). The sCCA model is expressed as, u^T^X^T^Yv subject to $\|\text{u}{\|}^{2}\leq 1,\|\text{v}{\|}^{2}\leq 1,{\text{P}}_{1}(\text{u})\leq c_{1},{\text{P}}_{2}(\text{v})\leq c_{2},{\text{u}}_{\text{j}}=0\forall_{\text{j}}\notin {\text{Q}}_{1},{\text{v}}_{\text{j}}=0\forall_{\text{j}}\notin {\text{Q}}_{2}$ . X and Y denote the paired multi-omics datasets, u and v are the canonical vectors containing the weights for each feature, and Xu and Yv, taken to be the weighted linear combinations of features within each subject, are the canonical scores. The *P_1_* and *P_2_* represent lasso penalty functions on the canonical variates, and the resulting u and v are sparse for *c_1_* and *c_2_* sufficiently small. *Q_1_* and *Q_2_* denote subsets of features in X and Y that have large univariate correlation with the phenotype, and features that are not strongly associated with the phenotype are automatically assigned zero weights. The optimal tuning parameters for the model were selected by 10-fold cross validation.

### Inter-Omics Network Analysis

The sCCA selected microbes and metabolites were used as input to construct the inter-omics Gaussian graphical model (GGM), where the edges represent partial correlations between features. The optimal GGM was selected by minimizing the extended Bayesian information criterion (EBIC) of unregularized GGM models (Foygel and Drton, 2010). We first selected the top 100 models by estimating a sparse inverse covariance matrix along a path of regularization parameters using the graph lasso penalty to select the significant edges. Each of these models was refit without regularization, and the model with the smallest EBIC was chosen as the optimal network.

## Results

### Sample Characteristics

The sample consisted of 499 peri- and early post-menopausal Chinese women that provided both stool and blood samples for metagenomic and metabolomic profiling (**Table 1**). 84% of these women were classified as postmenopausal (i.e., >1 year since final menstrual period), while 16% were still in the perimenopause transition period. The average time since menopause was 2.0 years (SD = 1.0), corresponding to the life stage when women typically begin to experience rapid bone loss (Melton et al., 1992). On average, the subjects were 53.0 years old (SD = 2.9) with body mass index (BMI) of 23.0 kg/m^2^ (SD = 2.9) and reported exercising approximately once per week (SD = 0.8). 62% of the women had undetectable estradiol levels (<18.35 pmol/L), an indicator of menopause, and the average level of FSH was 76.2 mIU/ml (SD = 32.2). BMI, exercise, time since menopause, estradiol, and FSH had significant bivariate associations with BMD (*p*-values <0.05).

**Table 1:** Sample Characteristics (n = 499).

	mean (sd)/n (%)	*β*	*p*-value
Age	52.8 (2.9)	-0.01	0.42
Weight (kg)	57.3 (7.8)	0.05	<0.001
Height (cm)	157.9 (5.1)	0.05	<0.001
BMI (kg/m^2^)	23.0 (2.9)	0.11	<0.001
Family Income (Yuan)			
<60,000	129 (25.8%)	0.10	0.07
60,000 – 120,000	217 (43.5%)		
>120,000	153 (30.7%)		
Exercise (times/week)	0.81 (0.8)	0.13	0.02
Years Since Menopause	2.0 (1.0)	-0.13	0.003
Follicle Stimulating Hormone (mIU/ml)	76.2 (32.2)	-0.01	<0.001
Estradiol			
Low (<18.35 pmol/L)	308 (61.7%)	0.24	0.008
High	191 (38.3%)		
Calcium Supplementation			
Never	289 (57.9%)	-0.01	0.83
Sometimes	137 (27.5%)		
Often	73 (14.6%)		
Alcohol			
Sometimes Never	143 (28.7%) 355 (71.1%)	0.02	0.81
Smoking			
Sometimes	0 (0.0%)	–	–
Never	499 (100.0%)		
Femoral Neck BMD (g/cm^2^)	0.86 (0.12)	–	–

Means and standard deviations are provided for continuous variables. Number of subjects and percentages are provided for categorical variables. β and p-value provide the bivariate associations between each variable and BMD based on linear regression.

### Microbiome Association Analyses

After shotgun metagenomic sequencing of the stool DNA samples, we obtained approximately 7.35 giga base pairs of sequencing data per subject. Among >10,000 microbial features, there were 672 species with an average relative abundance >0.01%, which accounted for approximately 96% of the total microbiome across all subjects. 59.2% of these taxa belong to the *Firmicutes* phylum, 31.5% to *Bacteroidetes*, 6.1% to *Proteobacteria*, 2.2% to *Actinobacteria*, 0.5% to *Fusobacteria*, and 0.5% to *Verrucomicrobia*. On average, the *Bacteroidetes* and *Firmicutes* phyla accounted for 50% and 45% of the microbiome composition, respectively.

**Figure 2:**
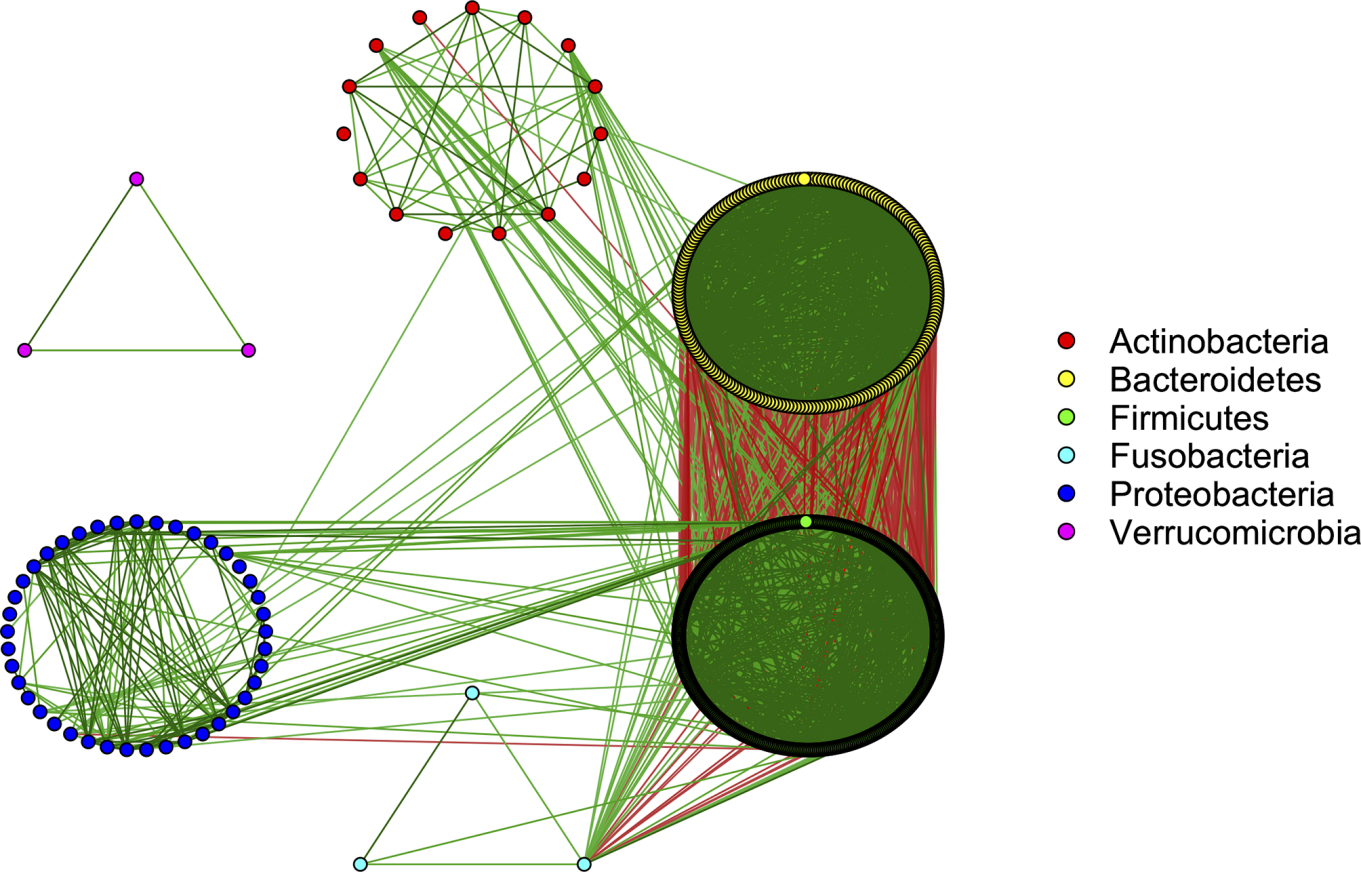
Co-occurrence network showing strong interactions in the microbiome community (SparCC correlations >0.5 and <-0.5). Green/red edges correspond to positive/negative relationships. The color of each node corresponds to the phylum of the given microbe.

The SparCC approach (Friedman and Alm, 2012) was applied to explore the strength of relationships between the microbiota. SparCC accounts for the compositional nature of the data by approximating the correlations between the log-ratio transformed relative abundances. We observed that 8,697 pairs of taxa had strong positive correlations (ρ’s > 0.5 and *p*-values < 0.001), while 898 had strong negative correlations (*ρ*’s < –0.5 and *p*-values < 0.001). These relationships were visualized by the microbiome co-occurrence network (**Figure 2**). The underlying causes of these microbial interactions are complex. While mutualistic and phylogenetically related bacteria may sometimes co-occur, this is not always the case. Similarly, microbes with antagonistic relationships, such as those competing for the same niche, may sometimes have inverse associations, while in other circumstances they may actually co-occur due to variation in their shared environment (Levy and Borenstein, 2013). Co-exclusions can also arise due to incompatible abiotic factors in the microbiome community (Weiss et al., 2016).

There were 44 taxa identified for potential association with BMD in the initial feature selection by the constrained elastic net regression model, including 22 which also had *p*-values <0.05 when tested individually using partial Spearman correlation analysis adjusted for relevant covariates (**Table 2**). Among the putative BMD associated microbes, 9 had FDR <0.05, and the remaining 13 had FDR <0.1. Several of the identified species including *Bacteroides vulgatus*, *Bacteroides uniformis*, *Bacteroides fragilis*, and *Bacteroides massiliensis*, all of which were negatively associated with BMD, were among the most abundant species in the microbiome with average relative abundances >1%. On the other hand, Firmicutes microbes, such as *Clostridium leptum* and *Ruminococcus lactaris*, were observed to be positively associated with BMD.

**Table 2:** BMD associated microbial species.

	ρ_adj_	P-value	FDR	Abundance	Phylum
Prevotella sp. BV3P1	-0.14	0.001	0.02	0.03%	Bacteroidetes
Prevotella disiens	-0.14	0.002	0.02	0.03%	Bacteroidetes
Bacteroides massiliensis	-0.14	0.002	0.02	1.06%	Bacteroidetes
Bacteroides fluxus	-0.13	0.003	0.02	0.15%	Bacteroidetes
Parabacteroides sp. D13	-0.13	0.003	0.02	0.15%	Bacteroidetes
Bacteroides sp. 2-1-56FAA	-0.12	0.007	0.04	0.03%	Bacteroidetes
Anaerotruncus sp. CAG:528	0.12	0.007	0.04	0.03%	Firmicutes
Odoribacter laneus	-0.12	0.008	0.04	0.02%	Bacteroidetes
Parabacteroides distasonis	-0.12	0.010	0.04	0.51%	Bacteroidetes
Clostridium leptum	0.11	0.015	0.05	0.05%	Firmicutes
Bacteroides oleiciplenus	-0.11	0.015	0.05	0.19%	Bacteroidetes
Fusobacterium ulcerans	-0.11	0.017	0.05	0.05%	Fusobacteria
Bacteroides fragilis	-0.11	0.020	0.05	2.58%	Bacteroidetes
Bacteroides uniformis	-0.10	0.020	0.05	2.76%	Bacteroidetes
Ruminococcus lactaris	0.10	0.021	0.05	0.26%	Firmicutes
Firmicutes bacterium CAG:341	0.10	0.024	0.05	0.05%	Firmicutes
Coprobacillus sp. CAG:235	0.10	0.027	0.06	0.11%	Firmicutes
Bacteroides vulgatus	-0.10	0.034	0.07	5.64%	Bacteroidetes
Bacteroides sp. 2-2-4	-0.10	0.033	0.07	0.25%	Bacteroidetes
Bacteroides sp. 4-3-47FAA	-0.09	0.038	0.07	0.41%	Bacteroidetes
Blautia sp. CAG:237	0.09	0.038	0.07	0.07%	Firmicutes
Bacteroides sp. 3-1-40A	-0.09	0.039	0.07	0.28%	Bacteroidetes

Correlation coefficients and p-values correspond to the effects of the microbes individually tested in partial Spearman correlation analyses. Associations are adjusted for age, BMI, exercise, years since menopause, FSH, and estradiol. FDR correction accounts for testing the subset of bacteria species selected in the initial feature screening by constrained elastic net regression. Abundance refers to the average relative abundance across all samples.

Functional profiling of the microbiome yielded pathway abundances for 516 metabolic pathways in the microbial community, all of which involve the microbiota producing metabolic byproducts from the catabolism of dietary components. Partial Spearman correlation analyses identified 22 pathways for putative association with BMD at a threshold of *p*-value <0.05 (**Table 3**). However, due to the number of pathways tested and the modest effect sizes, none of the pathway associations remained significant after multiple testing correction (FDR >0.2).

**Table 3:** BMD associated microbial metabolic pathways.

MetaCyc ID	Pathway	ρ_adj_	P-value
PWY-5941	Glycogen degradation II	0.13	0.005
PWY-5181	Toluene degradation III (via p-cresol)	0.13	0.006
PWY-6185	4-methylcatechol degradation	0.12	0.006
PWY-5695	Urate biosynthesis	–0.11	0.011
CATECHOL-ORTHO-CLEAVAGE-PWY	Catechol degradation to β-ketoadipate	0.11	0.012
PWY-5417	Catechol degradation III	0.11	0.012
PWY-5659	GDP-mannose biosynthesis	0.11	0.013
CRNFORCAT-PWY	Creatinine degradation I	0.10	0.021
GLYCOGENSYNTH-PWY	Glycogen biosynthesis I (from ADP-D-Glucose)	0.10	0.023
PWY-6182	Superpathway of salicylate degradation	0.10	0.024
GALACTUROCAT-PWY	D-galacturonate degradation I	0.10	0.026
PWY-1861	Formaldehyde assimilation II	0.10	0.030
PWY-3841	Folate transformations II	–0.10	0.032
HOMOSER-METSYN-PWY	L-methionine biosynthesis I	0.09	0.037
PWY-7374	1,4-dihydroxy-6-naphthoate biosynthesis I	–0.09	0.037
1CMET2-PWY	N10-formyl-tetrahydrofolate biosynthesis	–0.09	0.038
PWY-6703	PreQ_0_ biosynthesis	–0.09	0.040
ARGININE-SYN4-PWY	L-ornithine *de novo* biosynthesis	–0.09	0.040
P124-PWY	Bifidobacterium shunt	0.09	0.042
PWY-5347	Superpathway of L-methionine biosynthesis	0.09	0.042
CITRULBIO-PWY	L-citrulline biosynthesis	–0.09	0.043
METSYN-PWY	L-homoserine and L-methionine biosynthesis	0.09	0.043

ρ_adj_ denoted partial Speraman correlation coefficient with adjustment for age, BMI, exercise, years since menopause, FSH, and estradiol.

Predictive metabolomic profiling was performed to impute the fecal metabolite profiles based on the gene abundances in the microbiome communities. Among 80 predicted intestinal metabolites, 3 were identified for potential association with BMD based on VIP ≥2.0 in PLS, and 17 had *p*-values <0.05 with FDR of 0.2 when individually tested using linear regression (**Table 4**). Several of these compounds including butyrate, propionate, and valeric acid are short chain fatty acids (SCFAs), a special class of microbial byproducts that play an important role in gut and metabolic health (Blaak et al., 2020).

**Table 4:** BMD associated imputed fecal metabolites.

	VIP	β_adj_	P-value	FDR
Nicotinate	2.27	0.11	0.009	0.20
Deoxyinosine	1.82	0.10	0.015	0.20
N-oleoylethanolamine	1.75	-0.10	0.016	0.20
Linoleoyl ethanolamide	1.64	-0.10	0.018	0.20
Docosapentaenoate	1.45	-0.10	0.018	0.20
Xanthine	2.33	0.10	0.019	0.20
Docosapentanoic acid	1.44	-0.10	0.020	0.20
Butyrate	1.83	0.10	0.020	0.20
Propionate	2.52	0.09	0.025	0.22
C18:0 Monoacylglycerol	1.30	-0.09	0.036	0.22
Eicosatrienoic acid	1.31	-0.09	0.037	0.22
Stearoyl ethanolamide	1.12	-0.08	0.043	0.22
Glutamate	1.20	0.08	0.044	0.22
Valeric acid	1.47	0.08	0.046	0.22
C16:0 ceramide	1.16	-0.08	0.046	0.22
Bilirubin	1.37	-0.08	0.046	0.22
Adrenic acid	1.21	-0.09	0.047	0.22

VIP refers to the variable importance in projection score provided by PLS. β_adj_ and p-values correspond to the effects of metabolites individually tested in linear regression adjusted for age, BMI, exercise, years since menopause, FSH, and estradiol. Metabolite identities were validated in the original publication for the reference samples used to train the MelonnPan model (see methods).

### Serum Metabolite Association Analyses

Based on LC-MS untargeted metabolomics profiling of the serum samples, 3,202 unique metabolite features were identified in positive ion mode and 2,674 were detected in negative ion mode. Among the unique metabolite features, 381 had putatively confirmed identities. There were 12 serum metabolites identified for potential association with BMD based on VIP ≥2.0 in PLS, and 13 which had *p*-values <0.05 when tested individually by linear regression (**Table 5**). 8 serum metabolites were detected by both approaches, but none of the identified metabolites remained significant after the multiple testing correction (FDR >0.2). Notably, several putative BMD associated serum metabolites including 3-phenylpropanoic acid, which is primarily derived from the degradation of plant polyphenols (Trost et al., 2018), and glycolithocholic acid (Taylor and Green, 2018), a secondary bile acid, are intricately linked with the microbiota. While both these compounds were imputed in the fecal metabolite analysis, no significant associations were observed for the predicted fecal abundances.

**Table 5:** BMD associated serum metabolites.

	m/z	RT	VIP	β_adj_	P-value	FDR
Alpha-D-Glucose	179.05	192.7	2.83	0.13	0.003	0.92
Hippuric acid	178.05	192.7	2.10	0.10	0.017	0.92
Glycolithocholic acid	432.31	191.3	1.50	0.10	0.017	0.92
LysoPC (18:0)	524.37	40.6	2.14	-0.10	0.019	0.92
D-Ribose	149.04	303.0	1.70	0.10	0.020	0.92
3-Phenylpropanoic acid	149.06	83.5	1.95	0.10	0.022	0.92
Palmitic acid	315.25	41.2	1.94	-0.10	0.022	0.92
1,7-Dimethylxanthine	181.07	57.5	2.47	0.09	0.026	0.92
Dodecanoic acid	199.17	88.2	2.52	-0.09	0.028	0.92
Histidinyl glycine	195.09	36.8	2.77	0.09	0.028	0.92
Quinate	191.05	326.0	2.57	0.09	0.039	0.92
3-Hydroxydodecanoic acid	431.34	36.6	2.07	-0.09	0.033	0.92
DAG (18:0/20:4)	627.53	189.3	1.90	-0.09	0.042	0.92
PC (18:0/18:1(9Z))	832.58	41.5	2.27	-0.08	0.057	0.92
Alpha-linolenic acid	277.21	37.2	2.03	-0.07	0.093	0.92
Urea	61.04	86.5	2.11	-0.07	0.120	0.92
2-Methyl-3-hydroxybutyrate	182.08	305.2	2.05	0.05	0.211	0.92

m/z denotes mass-to-charge ratio, RT indicates retention time. VIP refers to the variable importance in projection score provided by PLS. β_adj_ and p-values correspond to the effects of metabolites individually tested in linear regression adjusted for age, BMI, exercise, years since menopause, FSH, and estradiol.

### Multi-Omics Integration

**Figure 3:**
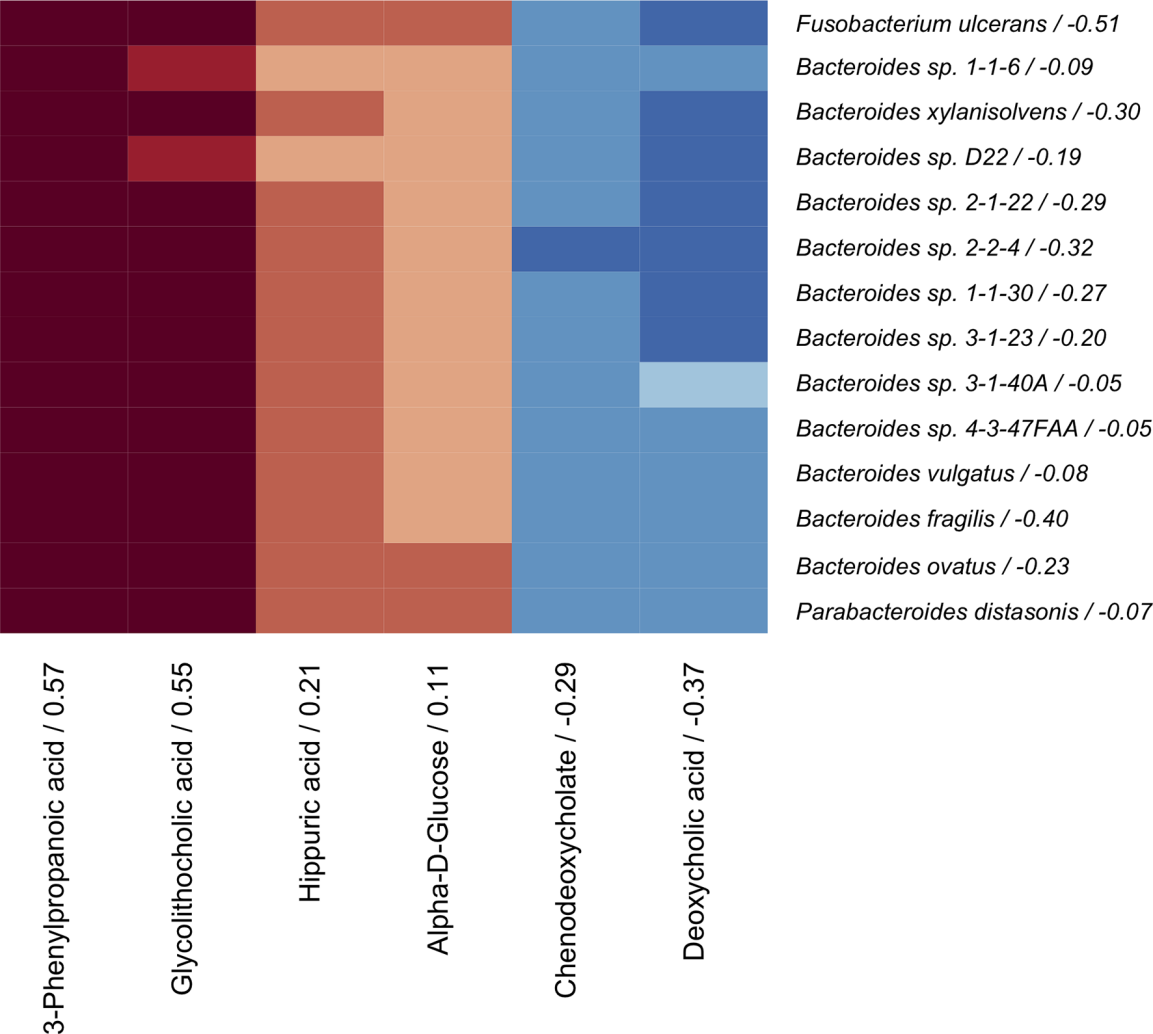
Correlation heatmap of sCCA selected microbes (rows) and metabolites (columns) that are correlated with each other and with BMD. Canonical loadings are provided for each feature, which represent the contributions to the inter-omics relationship. Positive correlations are represented by blue, negative correlations are shown in red, and intensity of the color represents the strength of association.

The coinertia analysis indicated that there were at least some statistically significant correlations between the microbiome and serum metabolome that should be further explored (RV = 0.064, *p*-value = 0.01). The sCCA model was then applied to the paired metagenomic and serum metabolite profiles, as has been done previously (Lee-Sarwar et al., 2019). We identified 14 bacteria species (*Bacteroides vulgatus*, *Bacteroides fragilis*, *Bacteroides ovatus*, *Bacteroides xylanisolvens*, *Parabacteroides distasonis*, *Bacteroides* sp. *4-3-47FAA*, *Bacteroides* sp. *1-1-6*, *Bacteroides* sp. *3-1-40A*, *Bacteroides* sp. *2-2-4*, *Bacteroides* sp. *3-1-23*, *Bacteroides* sp. *1-1-30*, *Bacteroides* sp. *D22*, *Bacteroides* sp. *2-1-22*, and *Fusobacterium ulcerans*) and 6 serum metabolites (3-phenylpropanoic acid, hippuric acid, alpha-D-glucose, chenodeoxycholate, deoxycholic acid, and glycolithocholic acid) that were correlated across omics modalities with importance for BMD (**Figure 3**).

**Figure 4:**
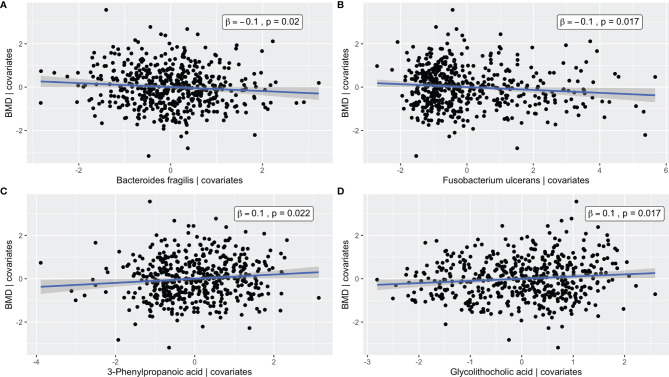
Added variable partial regression plots for **(A)** *Bacteroides fragilis*, **(B)** *Fusobacterium ulcerans*, **(C)** 3-Phenylpropanoic acid, and **(D)** Glycolithocholic acid. The plots illustrate the relationships between the abundance of a given microbe/metabolite and BMD after adjustment for age, BMI, exercise, years since menopause, FSH, and estradiol. The x-axes correspond to the residuals from regressing the microbe/metabolite on the covariates. The y-axes correspond to the residuals from regressing the phenotype on the covariates. The blue line represents the line of best fit from linear regression, and the corresponding confidence interval is shown in gray.

The metabolite and microbiome canonical scores, taken to be the weighted linear combination of features within each subject, were correlated with each other (*ρ* = 0.45, *p*-value < 0.001) and with BMD (*β_adj_* = 0.09 and 0.03 with *p*-values = 0.002 and 0.03, respectively), demonstrating the effectiveness of the supervised integrative feature selection. Based on the magnitude of the canonical loadings, which represent the contributions of each feature to the inter-omics relationship, the most important metabolites in the inter-omics relationship with respect to BMD were 3-phenylpropanoic acid and glycolithocholic acid, while the bacteria with the largest loadings were *Fusobacterium ulcerans* and *Bacteroides fragilis*, each of which were also detected in the single omics association analyses. The relationships between the abundances of these features and BMD after adjustment for covariates were visualized by added variable partial regression plots (**Figure 4**).

**Figure 5:**
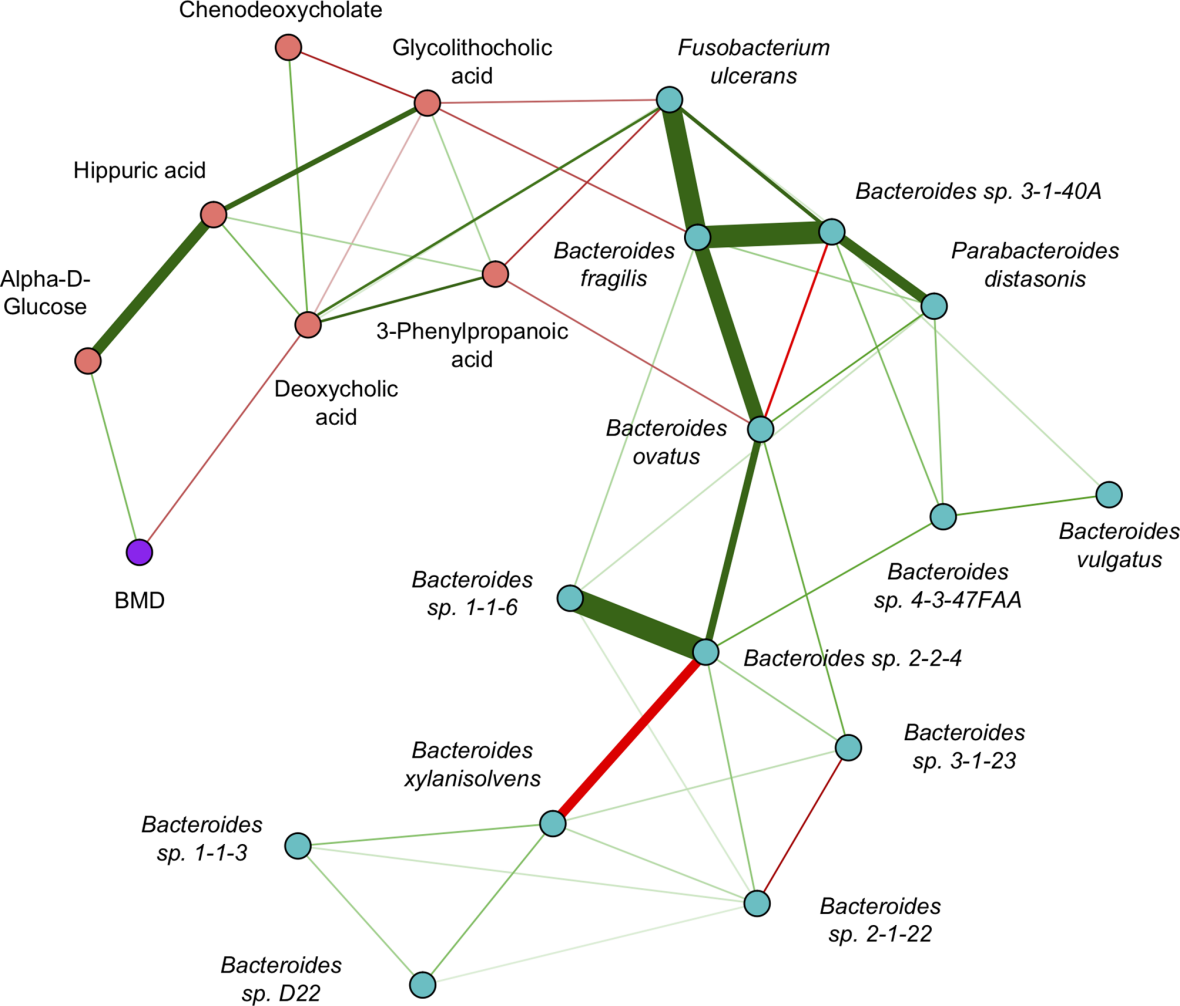
Inter-omics Gaussian graphical model for sCCA selected features. The edges represent partial correlations, and significant edges were selected by the graph lasso penalty. Blue/red nodes correspond to microbe/metabolite features, BMD is shown in purple, and green/red edges correspond to positive/negative partial correlations. The edge width indicates the strength of association.

The GGM (**Figure 5**) had an edge density of 0.22, which represents the ratio of the number of edges and the number of possible edges, and a transitivity of 0.46, which is defined as the probability that adjacent nodes of a given node are connected. *Fusobacterium ulcerans* was positively connected to deoxycholic acid and negatively connected to both 3-phenylpropanoic acid and glycolithocholic acid. *Bacteroides fragilis* was negatively connected to glycolithocholic acid, while *Bacteroides ovatus* was negatively connected to 3-phenylpropanoic acid. Alpha-D-glucose and deoxycholic acid were positively and negatively connected to BMD, respectively.

## Discussion

In this systematic multi-omics analysis of a relatively large sample of peri- and early postmenopausal Chinese women, we characterized the microbiota, serum metabolites, and possible crosstalk between these biological factors that may influence bone physiology. To our knowledge, this is one of the first reports to integrate paired metagenomic and metabolomic profiles to provide novel insights into the molecular mechanisms of skeletal remodeling. The findings, although biologically plausible, still require replication and functional validation in future studies.

Many of the putative BMD associated microbes belong to the *Bacteroides* genus, which were inversely associated with bone mass. *Bacteroides vulgatus* and *Bacteroides fragilis*, which were identified in both the single omics and integrative analyses, have previously been reported to induce activation of the pro-inflammatory NF-κβ signaling pathway, which is associated with bone loss (Kim et al., 2002). Additionally, *Bacteroides vulgatus* was recently shown to increase serum levels of the bone resorption marker CTX-1 and decrease serum levels of the bone formation marker osteocalcin *in vivo* in an ovariectomized (OVX) mouse model (Lin et al., 2020). On the other hand, we observed a positive effect for the *Firmicutes* microbe *Clostridium leptum*, a probiotic species that is known to be an important producer of beneficial metabolic byproducts such as butyrate (Canani et al., 2012). We further observed a negative effect of *Fusobacterium ulcerans*, which also played an important role in the multi-omics integration analysis. *Fusobacteria* have been shown to promote M1 macrophage production *via* AKT2 signaling (Liu et al., 2019), which induces inflammation and has been associated with the development of osteoporosis (Yang and Yang, 2019).

To investigate the potential mechanisms by which the microbiota may influence BMD, we profiled the abundances of metabolic pathways in the microbial community and assessed their associations with BMD variation. Although the pathway associations were not significant at a stringent threshold accounting for multiple testing, several were still interesting due to their known roles in bone metabolism. We observed a positive association between BMD and several glycolytic pathways, such as glycogen biosynthesis/degradation, which are essential for cellular energy (Adeva-Andany et al., 2016). We further identified a negative association for urate biosynthesis, and it has been reported that uric acid induces intracellular oxidative stress and inflammatory cytokines that stimulate bone resorption by osteoclasts and inhibit bone formation by osteoblasts (Austermann et al., 2019). Lastly, we detected a positive association for L-methionine biosynthesis, an amino acid which has been shown to down-regulate NF- κβ signaling in osteoclast precursors to reduce bone loss (Vijayan et al., 2014).

Since microbial metabolites contribute to host-microbiome interactions (Rooks and Garrett, 2016), we performed metabolomic imputation to predict the intestinal metabolite profiles based on the observed genes in the microbiome community. Similar techniques are frequently used in genetic association studies to impute the gene expression levels based on genotype information (Gamazon et al., 2015). We identified positive associations between BMD and several SCFAs, which are exclusively produced by the microbiota through the breakdown of non-digestible dietary fiber. The SCFAs are potent signaling molecules that modulate host gene expression by interacting with various epigenetic factors such as DNA methylation and histone acetylation (Alenghat and Artis, 2014). Butyrate and propionate have previously been reported to induce metabolic alterations of osteoclasts that lead to down-regulation of crucial genes such as TRAF6 and NFATc1 (Lucas et al., 2018). Butyrate has also been shown to stimulate bone formation through regulatory T cell mediated regulation of Wnt10b expression (Tyagi et al., 2018). Furthermore, valeric acid was recently demonstrated to promote/inhibit osteoblast/osteoclast differentiation *in vitro* (Lin et al., 2020). We note that the precision of fecal metabolite imputation by MelonnPan has room for improvement (Yin et al., 2020). Additionally, the prediction models were trained in a different study population (mixed sexes, North American, some with irritable bowel disease), and therefore the accuracy of the imputation in this sample is unknown.

While the fecal metabolites are the most representative of the direct metabolic output of the gut, many of those compounds are excreted from the body without ever having any influence on human health. The serum metabolome, which includes both host and microbiota derived metabolites, provides a window into which gut metabolic byproducts are absorbed into the circulating blood to potentially impact host physiology. We observed that metabolites involved in energy metabolism, such as alpha-D-glucose, were positively associated with BMD. Energy metabolism is critical for bone remodeling, and it has also been demonstrated that there is a feedback loop where bone can act as an endocrine gland by secreting bone specific proteins such as osteocalcin and osteoprotegerin, which can regulate insulin function and glucose metabolism (Faienza et al., 2015). Additionally, we detected the amino acid histidinyl glycine, a conjugation of histidine and glycine. Targeted deletion of histidine decarboxylase in mice, which converts histidine to histamine, was found to increase bone formation and protect against bone loss (Fitzpatrick et al., 2003). Glycine is reported to improve bone health by increasing the production of collagen, which is a major building block of bone (Jennings et al., 2016).

Several of the BMD associated serum metabolites are involved in lipid metabolism, and accumulating evidence has demonstrated that alterations in lipid levels are associated with changes in bone metabolism (Tian and Yu, 2015). Lipid metabolism is regulated PPARγ, which inhibits the differentiation of osteoblasts and promotes the formation of adipocytes (Wan, 2010). Dodecanoic acid was recently reported to have a causal effect on BMD, which was validated using bone cells cultured *in vitro* as well as *in vivo* in an OVX mouse model (Gong et al., 2021). Palmitic acid is a saturated fatty acid which has been shown to increase bone loss by promoting osteoclast survival (Oh et al., 2010). The increase of LysoPC (18:0), a lysophoshpatidylcholine, is indicative of oxidative stress, and LysoPCs have been detected at elevated levels in the serum of osteoporotic mice (Zhao H et al., 2018).

Most notably, the serum metabolite analysis identified several microbiota-linked compounds for association with BMD. 3-phenylpropanoic acid, also known as hydrocinnamic acid, is mainly produced by the microbial catabolism of dietary polyphenols, which are acquired from plant-based food sources such as leafy greens, tea/coffee, wheat, berries, fruits, and other vegetables (Trost et al., 2018). Dietary polyphenols have been reported to reduce the risk of various age-related diseases and have a lot of promise for protecting against bone loss due to their antioxidant properties (Sato et al., 2011). One study found that phenolic acids in the serum of rats fed a blueberry diet stimulated osteoblast differentiation, resulting in elevated bone mass (Chen et al., 2010). Additionally, *in vitro* experiments with phenolic acids using bone marrow stroma cells demonstrated stimulation of osteoblast differentiation and inhibition of adipogenesis (Chen and Anderson, 2014). Plant polyphenols are also an established source of Hippuric acid, which was recently observed to inhibit osteoclast formation *in vitro* (Zhao et al., 2020). On the other hand, Hippuric acid can also be derived from aromatic organic acids such as phenylalanine and tryptophan (Wikoff et al., 2009).

Glycolithocholic acid and deoxycholic acid are secondary bile acids that are formed when primary bile acids produced by the liver, such as cholic acid and chenodeoxycholate, enter into the intestine *via* the bile duct and are acted on by the microbiota (Taylor and Green, 2018). It has previously been reported that bile acids are essential for the intestinal absorption of lipids and lipid-soluble compounds such as vitamin D (Nehring et al., 2007), and abnormal bile acid turnover has been linked with osteoporosis in postmenopausal women (Hanly et al., 2013). Lithocholic acid and deoxycholic acid have been shown to enhance and reduce calcium absorption, respectively (Marchionatti et al., 2018). Additionally, a growing body of evidence suggests that bile acids may regulate skeletal remodeling processes through direct interactions with osteoblasts and osteoclasts (Cho et al., 2013).

The network analysis revealed several inter-omics relationships that could potentially play a role in the regulation of BMD. First, we observed a positive connection between *Fusobacterium ulcerans* and deoxycholic acid. Sulfate esterification of bile acids in the liver enhances their excretion (Alnouti, 2009), and *Fusobacteria* have been reported to be involved in desulfatation, which keeps them in circulation (Jia et al., 2018). Second, we observed a negative connection between *Fusobacterium ulcerans* and 3-phenylpropanoic acid. It has previously been reported that polyphenol compounds have antimicrobial properties that inhibit the growth and virulence of *Fusobacteria* (Ben Lagha et al., 2017). Third, we observed a negative relationship between *Bacteroides fragilis* and glycolithocholic acid. *Bacteroides* are reported to promote deconjugation of bile acids, and individuals with higher abundance of *Bacteroides* have been shown to have lower plasma levels of secondary bile acid metabolites (Gu et al., 2017). Lastly, we observed a negative connection between 3-phenylpropanoic acid and *Bacteroides ovatus*. Previous studies have demonstrated that dietary polyphenols can inhibit the growth of *Bacteroides* microbes (Cardona et al., 2013). However, further analyses are needed to determine the precise mechanisms of these relationships and how they may be relevant for bone physiology.

Despite the novelty of this study in the bone field, there are several limitations that should be taken into consideration. First, among thousands of unique metabolite features, we were only able to produce high confidence annotations for a relatively small number, and there could be important compounds in the serum, especially gut metabolites, that were ignored. Second, we have only considered linear relationships between the molecular features. In the future, this could be addressed through nonlinear extensions of the applied methods, which may capture complex statistical dependencies that conventional correlation approaches fail to detect (Szekely et al., 2007). Third, we can only speculate about the directionality of the inter-omics relationships observed in the network based on previously reported findings. Lastly, it is unclear if the findings can be generalized to other populations. Different ethnicities have vastly different diets, and metabolomic patterns are influenced by various factors including age, genetics, and menopause (Auro et al., 2014).

In summary, we conducted a comprehensive multi-omics integration analysis and provided novel insights into the interactions between the gut microbiome and serum metabolome that may be relevant for the regulation of BMD. We hope that these findings will stimulate future studies to further explore the relationships between the microbiome and host omics factors that may be involved in bone health.

## Data Availability Statement

The datasets presented in this study can be found in online repositories. The names of the repository/repositories and accession number(s) can be found below: https://www.ebi.ac.uk/metagenomics/, PRJEB50761.

## Ethics Statement

The studies involving human participants were reviewed and approved by Medical Ethics Committee of Southern Medical University. The patients/participants provided their written informed consent to participate in this study.

## Author Contributions

JG conducted the data analysis and prepared the manuscript. XL, K-JS, and RG contributed to the data analysis plan. HS contributed to the manuscript revision. JS and H-MX managed and directed the study components conducted within their respective institutions. H-WD conceived, designed, and directed the whole project. All authors contributed to the article and approved the submitted version.

## Funding

This research was benefited by the partial support by grants from the National Institutes of Health (P20GM109036, R01AR069055, U19AG055373, R01AG061917), the Science and Technology Program of Guangzhou, China (201604020007), the National Natural Science Foundation of China (81770878), and the National Key R&D Program of China (2016YFC1201805 and 2017YFC1001100).

## Conflict of Interest

The authors declare that the research was conducted in the absence of any commercial or financial relationships that could be construed as a potential conflict of interest.

## Publisher’s Note

All claims expressed in this article are solely those of the authors and do not necessarily represent those of their affiliated organizations, or those of the publisher, the editors and the reviewers. Any product that may be evaluated in this article, or claim that may be made by its manufacturer, is not guaranteed or endorsed by the publisher.
